# Engineering Breast Cancer On-chip—Moving Toward Subtype Specific Models

**DOI:** 10.3389/fbioe.2021.694218

**Published:** 2021-06-23

**Authors:** Carmen Moccia, Kristina Haase

**Affiliations:** European Molecular Biology Laboratory, European Molecular Biology Laboratory Barcelona, Barcelona, Spain

**Keywords:** breast cancer, microfluidics, tumor microenvironment, tumor-on-chip, preclinical model

## Abstract

Breast cancer is the second leading cause of death among women worldwide, and while hormone receptor positive subtypes have a clear and effective treatment strategy, other subtypes, such as triple negative breast cancers, do not. Development of new drugs, antibodies, or immune targets requires significant re-consideration of current preclinical models, which frequently fail to mimic the nuances of patient-specific breast cancer subtypes. Each subtype, together with the expression of different markers, genetic and epigenetic profiles, presents a unique tumor microenvironment, which promotes tumor development and progression. For this reason, personalized treatments targeting components of the tumor microenvironment have been proposed to mitigate breast cancer progression, particularly for aggressive triple negative subtypes. To-date, animal models remain the gold standard for examining new therapeutic targets; however, there is room for *in vitro* tools to bridge the biological gap with humans. Tumor-on-chip technologies allow for precise control and examination of the tumor microenvironment and may add to the toolbox of current preclinical models. These new models include key aspects of the tumor microenvironment (stroma, vasculature and immune cells) which have been employed to understand metastases, multi-organ interactions, and, importantly, to evaluate drug efficacy and toxicity in humanized physiologic systems. This review provides insight into advanced *in vitro* tumor models specific to breast cancer, and discusses their potential and limitations for use as future preclinical patient-specific tools.

## Introduction

Breast cancer (BC) is a predominant contributor to high annual rates of cancer-related mortality (11.6%), and thus the development of new breast cancer treatments is an essential priority (Bray et al., 2018). A recent review reported that the US FDA approved a mere 34 drugs between 1949 and 2018, with only 16 specific to BC (Leo et al., 2020). Triple negative breast cancer (TNBC), which is the most aggressive subtype, only has one specific targeted treatment option—a combination of chemotherapy and an immune checkpoint inhibitor (Atezolizumab), approved by the FDA in 2019 (Mavratzas et al., 2020). Following success in the preclinical phase, most oncologic drugs fail during clinical trials, with only 6.7% of those moving beyond Phase 1 (Hay et al., 2014). This fact underscores the weakness of current preclinical models—they simply do not accurately predict drug efficacy and toxicity in humans (Hay et al., 2014). Recent advances in engineered human *in vitro* models may improve the predictability of current treatments and benefit the drug development pipeline, and, in our opinion, should be added to the preclinical toolbox.

To predict their efficacy, it is necessary to study drugs within a physiologic environment—and for breast cancers this is particularly important (Xiao et al., 2019). Alongside complex drug interactions, a basic understanding of tumor development within the breast is still required. For example, it is still unclear how the tumor microenvironment (TME) contributes to the transformation and development of breast *in situ* carcinoma to an invasive form, and which microenvironmental aspects contribute to metastasis (Cowell et al., 2013). Moreover, how hormone fluctuations, during the menstrual cycle or pregnancy, contribute to changes in the breast tumor and TME are still relatively under-studied. Understanding these pivotal points in breast tumor development are crucial for developing efficient drug targets. Cancer cell lines and mouse models have provided invaluable contributions to the field of breast oncological research, having contributed to our current understanding of the genetic and mechanistic basis of BC development, as well as drug discovery and testing. While these methods will remain important for efficient large-scale screens, 2D cultures are physiologically dissimilar to patient-specific human tumors, and, in the case of animal models, dissimilar immune interactions and inherent heterogeneity denotes the lack of precise control over these systems.

To overcome these limitations, there has been a significant push toward the development of 3D models that accurately reflect the *in vivo* situation (Yamada and Cukierman, 2007). Tools, such as microfluidics, 3D printing, and organoids, are now commonly employed to develop more physiologic human tumor models (Trujillo-de Santiago et al., 2019). By growing cells in pre-defined architectures, and influencing tumor-microenvironment interactions, the integration of 3D co-cultures in microfluidic devices, so called tumor-on-chips, can recreate complex cell-cell and cell-matrix interactions in a dynamic, but highly controlled environment (Trujillo-de Santiago et al., 2019). Co-cultures of tumor-like BCs with various tissue-specific cells (fibroblasts, endothelial, and/or immune cells) are now commonly integrated in defined geometries, which can mimic breast-specific tissue regions. Although breast-specific models of this nature are in their infancy, we expect that, as they are further refined in the coming years, these models will contribute to the fundamental understanding of breast cancer development and may reveal new targets for treatment.

In this review, we critically examine recent advances in tumor-on-chip technologies with a specific focus on organ-mimicking structures, developed specifically for examining breast cancers. These models have been used to study the behavior of breast cancers and their interactions with the surrounding TME, and to examine drug efficacy and screen new compounds. Characteristics and classifications of different breast cancer subtypes are outlined, with an emphasis placed on the heterogeneity of each disease and tumor microenvironment. Recent tumor-on-chip devices, aimed at understanding specific breast cancer subtypes, are highlighted, noting their advancements and remaining challenges. Finally, we discuss the potential for use of these new *in vitro* 3D tools, their limitations, and highlight possible directions for their use as preclinical models. Breast cancer research and drug development can benefit from tumor-on-chip technologies that have the potential to bridge-the-gap between animals and humans. These new tools will hopefully lead to efficient, inexpensive, and robust means for studying patient-specific subtypes of this widespread disease.

## Classification of Breast Cancer

**FIGURE 1:**
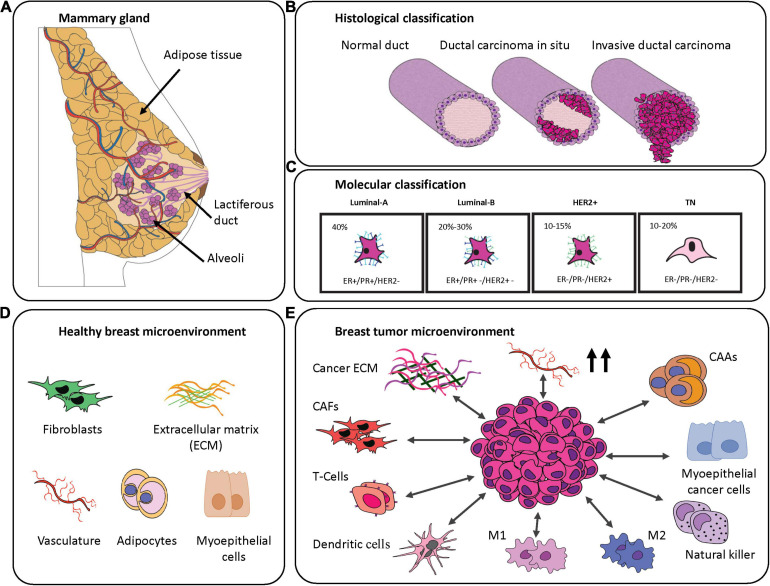
Breast cancer classification and breast-specific tumor microenvironment. **(A)** Graphical representation of the mammary gland. **(B)** Histological classification of the breast cancer subtypes. Magenta represents cancerous cells. **(C)** Molecular classification of the breast cancer subtypes demonstrating their frequency and commonly associated markers. **(D)** Schematic representation of components of the most abundant healthy mammary gland microenvironment. **(E)** Components often transformed in the breast tumor microenvironment.

The female breast develops as an apocrine gland specialized in the production and secretion of milk, which is rich in nutrients and antibodies to sustain offspring in the first days of life. The functional component of the breast is glandular and organized in a small unit, termed the mammary alveolus, which is lined with milk-secreting cuboidal cells surrounded by myoepithelial cells. In each breast, there are between 10 and 100 mammary alveoli that cluster into a lactiferous lobule (Hassiotou and Geddes, 2013). During and post-pregnancy, in response to stimuli from the hormones prolactin and progesterone, milk is produced and delivered from the lobules by the lactiferous ducts—tiny vessels lined with luminal epithelial cells (Pillay and Davis, 2020). The glandular tissue is embedded in a fibrous stroma composed of connective tissue, extracellular matrix (ECM), adipocytes, endothelial cells, fibroblasts and immune cells. While support and protection are offered by the stroma, the vascular and lymphatic systems supply oxygen, nutrients and control waste, respectively, to the glandular tissue (Insua-Rodriguez and Oskarsson, 2016; Figure 1A). Given the complex, dynamic, and hormonally regulated tissue structure of the breast, it is no surprise that a variety of BC subtypes can occur.

Considering its worldwide prevalence among women (Bray et al., 2018), BC requires clear classification in order to select the most appropriate therapy (Eliyatkin et al., 2015). This heterogeneous disease is characterized by differences in origin (specific structures within breast tissue), invasiveness (tumor cell infiltration), tumor grade (appearance), lymph node status (spread), and the presence of known predictive markers. A first classification evaluates the invasiveness of the BC cells based on histopathological analysis. From this first classification, BC is divided into the following categories (Figure 1B):

• **Non-invasive carcinoma** includes BCs with no spreading to the surrounding tissues. Cancerous cells remain confined in the lobular-duct system, giving rise to the most common types of BC, that is Ductal *In Situ* Carcinoma (DCIS) or to Lobular *In Situ* Carcinoma (LCIS) (Malhotra et al., 2010).
• **Invasive carcinoma** is defined by the fact that the cancer cells manage to invade into the surrounding tissues. If the tumor is derived from the DCIS it leads to Invasive Ductal Carcinoma (IDC), or more rarely, if it arises from LCIS it becomes Invasive Lobular Carcinoma (Malhotra et al., 2010).

Invasive carcinomas are further classified by grades that are scored on the level of differentiation of the cancer cells by evaluation of parameters like the mitotic rate, nuclear pleomorphisms, and the percentage of non-cancerous tissue (Rakha et al., 2008). Furthermore, it is possible to divide BC into five stages based on the tumor size, lymph-node involvement and whether any metastasis has occurred. There is a correlation between the higher stages of diagnosis and poor treatment outcome (Plichta, 2019). Another important factor that needs to be considered prior to treatment is the receptor status. Some breast cancers are well-known to overexpress different receptors including: estrogen receptor (ER), progesterone receptor (PR) and human epidermal growth receptor 2 (HER2) (Eliyatkin et al., 2015). These receptors enhance cell cancer growth, and since they are well characterized, it is possible to use anti-hormonal or anti-HER2 therapies, respectively, to treat these BC subtypes. Receptors can be used to define a molecular classification, based on immunohistochemistry features, which are divided into four major classes: luminal-A, luminal-B, and HER2^+^ carcinomas and triple negative breast cancer (TNBC) (Eliyatkin et al., 2015; Figure 1C). Luminal-A subtype is characterized by a high expression of ER and PR, while it is negative for HER2. It is the most common among the breast cancer subtypes and it is associated with a highly favorable prognosis. Due to the presence of hormone receptors, the patients benefit from endocrine therapies and aromatase inhibitors (Fragomeni et al., 2018). Luminal-B subtype is responsible of 20–30% of breast cancer cases and is characterized by a positive overexpression of ER with a variable presence of PR and HER2. It is associated with a more intermediate prognosis compared to Luminal-A, and often patients do not benefit from hormone therapy. HER2^+^ subtype, with a prevalence of 10–15% of total breast cancer cases, is characterized by amplification and elevated expression of the HER2 protein. Luminal-B BC is associated with higher tumor grades and a poor prognosis (Nascimento and Otoni, 2020). The remaining 10–20% of all diagnosed breast cancers are categorized as triple negative for their lack of expression of the three receptors: ER, PR, and HER2. TNBC is more often associated with hereditary conditions and patients with BRCA1 and/or 2 mutations are characterized by highly proliferative tumors, high tumor grade, risk of relapse and overall a poor prognosis (Nascimento and Otoni, 2020). Gene expression profiling of TNBCs shows a high heterogeneity of the disease and so TNBCs are often also classified by their molecular characteristics (Mehanna et al., 2019). Based on the classification proposed by Burstein (Burstein et al., 2015), TNBCs are subdivided in the following categories:

• Basal-like type 1 and 2, which are the most common types (75% of total cases), both characterized by a basal pattern of gene expression, but with two different immune response mechanisms;
• Luminal androgen receptor subtype is defined by a differential gene expression in the androgen metabolism pathway;
• Mesenchymal and mesenchymal stem-like subtypes are both characterized by the upregulation of genes involved in epithelial-mesenchymal transition, but differ in their expression of genes associated with stem cell and angiogenic factors.

Except for patients with BRCA1 and 2 mutations that could be treated by Poly ADP-ribose polymerase (PARP) inhibitors, platinum-based chemotherapy is typically selected as a treatment (Burstein et al., 2015). All of the aforementioned classifications are needed in order for clinicians to select appropriate treatments. Moreover, understanding the nuances of each BC classification is also indispensable for research into new treatments.

## Key Components of the Breast Tumor Microenvironment

Growth and progression of breast cancer is largely influenced by the niche where the tumor develops (Quail and Joyce, 2013). Specifically, the TME has been shown to play a role in the initiation, development, invasion and metastasis of cancer cells, and for this reason it is important to study tumor behavior within a native-like environment (Quail and Joyce, 2013). For BCs, this environment is characterized by the presence of organ-specific cell types including adipocytes and myoepithelial cells, stromal cells, and the vascular and immune systems. In this section, we discuss the role of key TME constituents and how they might contribute to BC tumor progression or inhibition (Figures 1D,E).

### Fibroblasts

One of the major players in the BC TME are fibroblasts—stromal cells responsible for supporting tissues by secreting proteins and remodeling the ECM. In homeostatic conditions, fibroblasts are inactive and quiescent, but their physiologic activation in the breast correlates with involution of the mammary gland after weaning. This activated state has been shown to be pro-tumoral (Guo et al., 2017), and these fibroblasts are often referred to as cancer-associated fibroblasts (CAFs). There is no clear definition of CAFs due to the uncertainty about their origin and expression of defining markers (Friedman et al., 2020). However, several markers including: fibroblast activation protein alpha (αFAP), fibroblast specific protein 1 (FSP-1), vimentin, and alpha smooth muscle actin (αSMA), are associated with upregulation of a myofibroblast-like phenotype (Friedman et al., 2020) and have been used as a proxy for CAF identification. According to various studies (Gascard and Tlsty, 2016; Gieniec et al., 2019; Louault et al., 2019), the percentage of CAFs in breast cancer could amount to 70% of the whole breast tumor volume. CAFs are thought to promote tumor progression by secreting pro-tumorigenic factors such as chemokines and matrix metalloproteinases (MMPs), which can induce stemness, genetic, and epigenetic changes in cancer cells, ultimately promoting metastatic events (Lugo-Cintrón et al., 2020). CAFs are heterogeneous and can be divided into subpopulations for different BC subtypes; four populations have been categorized by the different levels of expression of several markers including FAP, integrin β1/CD29, αSMA, FSP1, PDGFRβ, and CAV1. Luminal-like tumors are characterized by a significant presence of a CAF-S2 subpopulation, the same found in healthy breast tissue, suggesting that these CAFs are derived from normal resident fibroblasts. For the TNBC subtype, a high presence of immunosuppressive CAFs, defined as CAF-S1 and CAF-S4, have been shown to enhance the capacity of T-regulatory lymphocyte inhibition of effector T-cell proliferation (Costa et al., 2018). In particular, high levels of these two subtypes (S1 and S4), when diagnosed in the lymph node, are prone to develop late distant metastases, and thus could be used as prognostic markers (Pelon et al., 2020).

### Breast Extracellular Matrices

Inextricably linked to the remodeling and deposition of proteins by fibroblasts and other stromal cells, is the ever-changing ECM. The ECM is composed of collagens, proteoglycans, fibronectin, laminin and elastin, all produced by different mesenchymal cell types, including fibroblasts and endothelial cells (Kusuma et al., 2012). The ECM provides scaffolding and support to cells, to regulate cell signaling, as well as cellular processes like adhesion, migration, apoptosis, proliferation and differentiation (Velaei et al., 2016). The breast ECM is specifically composed of type IV collagens, laminin like LM-111 and LM-332, fibronectin, and several glycoproteins like hyaluronan (Oskarsson, 2013). Breast ECM composition drastically changes in response to hormonal stimuli during pregnancy, lactation, and post-lactation events (Oskarsson, 2013). For instance, the involution process of the mammary gland results in activation of fibroblasts and significant deposition of a fibrotic-like ECM, mainly composed of fibrillar collagens. Fibrillar collagens contribute to increased breast density, which is a known risk factor for BC in women, particularly since collagen organization contributes to tumor cell migration (Guo et al., 2017). The involution process also involves immature immune-cell infiltration (myeloid cells, M2-type macrophages, and T-cells) and extensive ECM remodeling, which could contribute to a pro-tumorigenic environment. As would occur during wound healing, there is a significant upregulation of fibrillar collagens, fibrillin, and increased proteolysis, which acts as a chemoattractant for macrophages (Insua-Rodriguez and Oskarsson, 2016). As well, the fragmentation of the ECM, by metalloproteases and remodeling enzymes, facilitates the penetration of CAFs and promotes the invasive behavior of tumor cells (Gkretsi et al., 2015). Thus, the dynamic changes in the ECM of the mammary gland is a cyclic process which can make this tissue more prone to dysregulation, thus supporting cancer development. Each breast cancer subtype presents a different and dynamic composition of the ECM, providing an optimal niche for tumor cells to thrive. For the most aggressive forms, like TNBC and HER2^+^, increased stiffness corresponds to significant collagen linearization and deposition that leads to enhanced immune cell infiltration. In contrast, luminal-like breast cancers undergo less ECM remodeling and are typically less-stiff, thus consistently less immune infiltrate and pro-invasion signaling occurs in these subtypes (Acerbi et al., 2015; Tang et al., 2018).

### Vasculature

Besides the stroma, the vasculature is another key player in the TME—since it is majorly responsible for delivery of critical growth factors, immune cells, and for providing a physical barrier to metastasis (Fang et al., 2014; Xie et al., 2017). Tumors necessitate a higher capacity of nutrients and oxygen, which corresponds with their increased release of pro-angiogenic factors like VEGF, leading to subsequent recruitment of endothelial cells and/or the co-option of existing vasculature (Maishi and Hida, 2017). Intratumorally, excessive VEGF expression contributes to the disordered formation of vessels which are excessively leaky, likely contributing to inefficient drug administration and shielding against the immune system (Klein, 2018). It has also been shown that tumor-associated endothelial cells present an altered phenotype and are more pro-angiogenic (Klein, 2018). Due to cytogenetic abnormalities, endothelial cells have a high chance of having multiple copies of drug-resistant genes transforming them into drug-resistant cells (Hida et al., 2018), which could also contribute to multiple drug resistance. Tumors significantly exploit vascular networks via their co-option and by altering endothelial barrier properties in order to progress through the key stages of metastasis (local invasion, intravasation and finally extravasation). As for many other types of cancer, angiogenesis influences breast cancer development and is a prognostic factor for the outcome of the patient (Bujor et al., 2018). In fact, for basal-like BC patients, an increased level of VEGF-C and D isoforms in the cancer cells and dense lymphatic networks are associated with aggressiveness and risk of metastasis (Madu et al., 2020).

### Immune Cells

Whether the immune system contributes to tumor growth or suppression is still a matter of debate (Hanahan and Coussens, 2012). Macrophages, dendritic cells, natural killer (NK) cells, myeloid-derived suppressor cells, and T-cells have all been shown to contribute to both, the inhibition, or promotion of tumor growth and survival (Hanahan and Coussens, 2012). Within highly inflammatory BC TMEs, immune infiltration is correlated to the presence of tumoral hormone receptors, and is variable for ER^+^, HER2^+^, and TNBC (Segovia-Mendoza and Morales-Montor, 2019). The most abundant immune cells associated with BCs are tumor associated macrophages (TAMs). TAMs differentiate from circulating monocytes and are classified as the classic M1 (pro-inflammatory), or activated M2 (anti-inflammatory) subtypes. On one hand, M1 TAMs recognize pro-inflammatory cytokines such as interferon-γ and are associated with anti-tumoral characteristics. On the other hand, M2 TAMs, which are typically associated with tissue repair and angiogenesis, demonstrate pro-tumorigenic characteristics and can arise in response to anti-inflammatory and regulatory cytokines, such as transforming growth factor beta-1 (TGF-β1) and interleukins (IL-4, IL-10 and IL-13) (Belli et al., 2018). In the breast TME, a higher infiltration of M2-like TAMs is correlated with unfavorable clinical features, such as increased tumor size, higher histological grade, ER negativity, and lower patient survival rates (Soysal et al., 2015). Other immune cells also contribute greatly to the TME. For instance, NK cells and neutrophils are strongly associated with ER positive BCs. And while typically less abundant, T-cell recruitment is associated with poor prognosis, regardless of receptor status (Segovia-Mendoza and Morales-Montor, 2019).

### Adipocytes and Adipose-Derived Stromal Cells

In addition to, and in concert with, immune cells are the presence of breast adipocytes that provide support and protection to the glandular tissue. Breast adiposity depends on a number of factors (mammary density, menopause status, and body mass) and can also change with the dynamics of breast tissue remodeling. For example, during involution macrophages are known to play a key role in adipogenesis (Soguel et al., 2017). The adipocytes in the breast TME are characterized by reduced lipid content, expression of specific adipokines, highly expressed proteases to degrade the ECM, and increased pro-inflammatory cytokine production. Of all the adipokines expressed, adiponectin is the only one known to support anti-tumoral activity (Jasinski-Bergner and Kielstein, 2019). In fact, a number of adipocytokines, including IL-6, TNF-α and VEGF, can promote the induction of CAFs and fibroblast-like endothelial phenotypes. These signaling changes promote the invasion and metastasis of BCs (Wu et al., 2019). Certain conditions, including obesity, can reversibly contribute to expression of these factors through chronic inflammation (Chu et al., 2019). Moreover, tumors themselves have been shown to dysregulate the metabolism of fatty acids and cause lipolysis in order to provide nutrients for their growth (Koundouros and Poulogiannis, 2020). Tumor-associated adipocytes have also been shown to secrete an altered spectrum of proteins. For example, overexpression of the proteoglycan versican, which can contribute to a tumorigenic environment, promotes angiogenesis, and could favor vascularization of solid BCs has been observed (Asano et al., 2017). Recently, Yang and colleagues published a microfluidic platform containing vascularized healthy adipose tissue formed with primary cells (Yang et al., 2020); however, the integration of adipocytes in a breast tumor-on-chip models has not yet been reported.

Adipose-derived stromal cells (ASCs) constitute another important, heterogeneous, group of cells present in normal breast adipose tissue. ASCs surround mature adipocytes and have the ability to differentiate into mature adipocytes *in vivo*, as well as into multiple mesenchymal lineages in response to alterations in the ECM, sex-specific hormones, and increasing body mass index (BMI) (Gimble et al., 2013). The role of ASCs in promoting breast cancer growth within the TME has been highlighted by several studies that recently showed a link between obesity and ASC phenotype (Gimble et al., 2013; Hillers et al., 2018). ASCs lose their capacity to differentiate into adipocytes and osteoblasts, presenting a myofibroblast lineage that may promote the rapid growth of invasive breast tumors in obese women (Hillers et al., 2018). This loss of differentiation potential, of ASCs in obesity, may result from exposure to chronic inflammatory signaling by macrophages recruited to adipose tissue (Hillers et al., 2018). A patient’s BMI should be considered in personalized treatments, and may have a critical impact on predicting drug efficacy in future *in vitro* model systems.

### Myoepithelium

Acting as the intermediary between the terminal ducts and lobules, surrounding stroma and adipose tissue, are myoepithelial cells. Normally, myoepithelial cells divide and protect the luminal breast epithelium by producing a basement membrane (BM) (Pandey et al., 2010). Myoepithelial cells can be considered a natural tumor-suppressor, since they prevent the spread of cancer cells from the lumen to the surrounding tissue via a physical barrier. Moreover, these cells secrete molecules such as nexin II, α1-antitrypsin, metalloproteases 1, thrombospondin-1, that block the invasive behavior of tumor cells, angiogenesis and BM degradation (Deugnier et al., 2002). On the other hand, the tumor microenvironment leads the myoepithelial cells to acquire genetic, cytogenetic and epigenetic mutations (Gudjonsson et al., 2005), which has contributed to the uncertainty of their role in metastasis. In fact, cancer cells need to disrupt and bypass the myoepithelial barrier in order to exit the mammary duct. There are two possible (and debated) scenarios surrounding how this phenomenon might occur. In one scenario, the cancer cells over-produce proteolytic enzymes such as matrix metalloproteinases (MMPs), serine proteases and cathepsins, that degrade the myoepithelium and allow the cells to invade into the surrounding stroma (Duffy et al., 2000). On the other hand, different studies support the hypothesis that damaged myoepithelial cells secrete various molecules that alter the microenvironment, induce an immune response, and also disrupt its own barrier (Singer et al., 2002; Man, 2007). Once the barrier integrity is compromised, tumor cells can cross the BM and come in contact with the stroma in order to further colonize the region. Regardless of the pathway, dysregulation of the myoepithelial cell barrier needs to be explicitly considered to further understand their role in the BC TME and the metastatic process, which could be done in a controlled way on-chip.

Given that breast tissue is composed of specific cell types and undergoes dynamic shifts in hormones, and that BC tumor progression is specific to its origin, we strongly support the importance and inclusion of the TME in *in vitro* model development. Models incorporating a relevant tumor niche will provide a deeper understating into which targets (potentially those of the TME, in addition to the tumor) will be useful for halting, and hopefully eliminating, BC tumor progression (Shang et al., 2019). For this reason, there is an urgent need to find suitable models able to mimic these complex TME interactions for the varied BC subtypes.

## Toward Tissue-Specific Breast Cancer Models

How close are we to producing reliable models for studying breast cancer? *In vivo* and *in vitro* models have certainly become more complex, with patient-specific models employed more regularly; however, there is still room for improvements in predicting drug efficacy, particularly for new drug development. Recapitulating the stages of cancer progression, complexities of the tumor microenvironment, as well as tissue-specific and heterogeneous tumor characteristics, are all aspects needed to be taken into consideration in model development. With this focus in mind, state-of-the art models for investigating BCs are discussed below.

### Animal Models of Breast Cancer

Animal models have provided tremendous contributions toward our understanding of cancer and relevant therapies. Sequencing of the mouse genome has made it possible to modify specific targets in mice—made to overexpress, or silence, particular genes at specific time points, in specific organs (Ireson et al., 2019). Genetically engineered modified mice (GEMMs) have been developed as preclinical tools for examining a number of drugs for breast cancer research. GEMMs utilize a mammary-gland-specific promoter, such as mouse mammary tumor virus (MMTV), or whey acidic protein (WAP), to confine the expression of the target gene in the epithelium of the mammary gland (Hennighausen, 2000; Sakamoto et al., 2015; Holen et al., 2017). Due to the spatial-temporal control of the tumor in GEMMs, it is possible to recapitulate tumor progression (classifiable by stage) and heterogeneity between animals, in order to validate candidate cancer genes. For example, To et al. (2014) generated GEMMs using the promoter MMTV to selectively delete the *BRCA1* and *p53* tumor suppressor genes, to closely resemble TNBC. In their study, GEMMs treated with different PARP inhibitors demonstrated chemo-preventive effects, which delayed tumor onset (To et al., 2014). These models have and continue to improve our understanding of cancer; however, they are limited by strain-specific genetic backgrounds and cannot fully recapitulate the human TME (Klinghammer et al., 2017).

To improve on the limitations of GEMMs, patient-derived xenografts (PDX) have been transplanted into immune-deficient mice, allowing researchers to maintain histologic and genetic features of the original tumors (Murayama and Gotoh, 2019). PDX mouse models are a valuable tool for precision medicine, since patient tumor-specificities are retained. Human stromal cells from xenografts are gradually replaced by murine stroma, proving that transplanted tumors retain the potential to recruit cells to their niche. PDX models have been created for all BC subtypes—as detailed in the review by Murayama and Gotoh (Murayama and Gotoh, 2019). Yet, there are still limitations of PDX models, including inconsistencies between human and murine stromal compartments, and the fact that they are developed in immunodeficient mice, making evaluations of the immune response negligible (Yoshida, 2020).

Transplantation of total peripheral blood or tumor-infiltrating lymphocytes (TILs) into immunodeficient mice, so-called humanized mice, have filled this gap of PDX and shed light on complex tumor-immune interactions *in vivo* (Lai et al., 2017; Stripecke et al., 2020). As an example, Rosato et al. explored the *in vivo* activity of a humanized anti-programmed cell death protein 1 (anti-PD-1) against TNBC established in a PDX model engrafted with human CD34^+^ hematopoietic stem cells (Rosato et al., 2018). The authors demonstrated that anti-PD-1 therapy results in a positive response to treatment, with a significant reduction in tumor growth and increased survival in some PDX. Interestingly, only the humanized mice responded positively, indicating the importance of the human immune system for the preclinical evaluation of immunotherapies in breast cancer (Rosato et al., 2018). For an overview of humanized mouse models in cancer research we suggest a further review by Tian et al. (2020a). While useful, these models (PDX with and without human immune cells) are slow to establish, are wide-ranging in success (10–80%, depending on the tumor type), and, similar to GEMMs, are expensive and labor-intensive (Pompili et al., 2016). These model systems are incredibly useful for studying tumors in an *in vivo* physiologic setting, yet animal models still pose a number of constraints limiting their ability to accurately predict the human response to cancer treatments. The drug development pipeline will likely continue to rely on animal models, but future pairing of these models with humanized *in vitro* systems may lead to improvements in the downstream outcome.

### Human Models of Breast Cancer

Besides animal models, cancer research has historically relied on the development of immortalized cancer cell lines and the use of 2D *in vitro* cultures. Cell culture in well-plates and with cell lines remains dominant in breast cancer research, since it provides a cost-effective, facile, and reproducible means to examine a large number of drugs (or other cell perturbations) simultaneously (Jensen and Teng, 2020). There are many established human BC cell lines available (Kondo and Inoue, 2019), many of which are accessible commercially and through cell biobanks. With their experimental reproducibility, low cost, and possibility of high-throughput analyses, cell lines provide an ideal solution for studying BC. Growing cells in a monolayer (on plastic or glass) is not representative of the physiology of the original tumor, as it forces cells to undergo modifications in polarity and shape due to homogenous access to nutrients, oxygen, and the exposure to drugs is also vastly different than *in vivo* (Hoarau-Vechot et al., 2018). Some of these limitations have been recently overcome using a variety of 3D culture techniques.

3D culture can be broadly categorized into non-scaffold and scaffold-based techniques (Jensen and Teng, 2020). Scaffold-free techniques allow cells to self-assemble into non-adherent cell aggregates (spheroids) through the use of low-adhesion plates, micropatterned surfaces, rotating bioreactors, magnetic levitation and 3D bioprinting (Langhans, 2018). Cell aggregates, or spheroids, are typically formed from either cancer cell lines or patient-derived samples (typically organoids, or PDOs). Spheroids mimic solid tissues by secreting their own ECM and by displaying differential nutrient availability, oxygen and drug distribution throughout the cell mass, thus providing a better prediction of drug response and/or resistance (Edmondson et al., 2014). Spheroids generated from cell lines are typically consistent in size and shape (depending on source) and therefore useful for high-throughput screening (Langhans, 2018). High-throughput compound screens have been performed using BC and non-cancerous spheroids, revealing promising tumor-targeting compounds including microtubule-targeting agents and epidermal growth factor inhibitors (Howes et al., 2014). Interestingly, this selective tumor targeting seen in spheroids could not be confirmed in 2D, underlining the importance of employing a 3D culture system to recapitulate complex *in vivo* drug interactions—a finding our previous work also supports (Haase et al., 2020). Besides cell lines, patient-derived samples or organoids have also been used to represent the heterogeneity of patient-specific tumors in 3D. Established biobanks of PDOs are now available (Sachs et al., 2018); however, some challenges are associated with PDO use, including the limited control over contamination of the biopsy with normal epithelial tissue, difficulty in maintaining tumor heterogeneity over time, and the lack of signaling from a native TME (Nagle et al., 2018).

The integration of either spheroids or PDOs with components of the TME (stromal, endothelial and immune cells) can provide a more physiologic-like environment for characterization of cancer progression and for examining TME-specific effects. Scaffold-based 3D cultures can provide these complex TMEs, by embedding tumor cell aggregates into a matrix from either natural (biological) or synthetically engineered sources—to mimic key properties of tissue-specific ECM properties (stiffness, electrical charge, or adhesiveness). An example is the use of a tri-component hydrogel using collagen, alginate and fibrin that has been developed as a functional ECM analog to mimic the stiffness of native soft tissues (Xu and Wang, 2015; Zhao et al., 2015; Zhou et al., 2016). Complex TMEs can be developed to limit diffusion-related barriers via vascular networks, as has been done recently using microfluidic technologies, like those used in ours (Haase et al., 2020) and other groups (Choi et al., 2015; Shirure et al., 2018; Aung et al., 2020; Nashimoto et al., 2020). For an extensive overview of 3D cell culture techniques, we refer the readers to reviews on these topics (Duval et al., 2017; Lv et al., 2017; Jensen and Teng, 2020).

Bioprinting techniques have also recently led to further exploration of interactions between the tumor and TME in a highly controlled manner (Bae et al., 2020). 3D bioprinting enables layer-by-layer construction of complex geometries of cells, ECM proteins and biomaterials with high spatial resolution (Datta et al., 2020). Thus, bioprinting can be used to develop cell-laden architectures that mimic target tissues or organs of interest (Cleversey et al., 2019; Bae et al., 2020). These techniques have been used to explore different aspects of BC research, including the generation of implants for breast tissue replacement (Cleversey et al., 2019) and the development of anti-cancer drugs for personalized therapies (Bae et al., 2020). An in-depth discussion is beyond the scope of this text, and so we refer the reader to another detailed review (Sharifi et al., 2021).

With advances in microfluidics, biomaterials, and bio-printing, tumor-on-chip systems allow for the study of complex cell-interactions in more relevant and highly controlled, engineered, TMEs. Bottom-up design approaches and semi-controlled cell behaviors will allow for complex BC model development. BC models with increased complexity have the potential to provide significant insight into subtype specific tumor development and progression, and should prove useful in predicting drug efficacy—both topics are covered in the following sections.

### Human Breast Tumor-on-Chip Models

The use of microfluidics in oncological research presents several advantages, including precise fluid handling, small sample requirements, cost-effectiveness, environmental control and high-throughput multiplexing capabilities (Minteer and Moore, 2006). Annually, there is a continued increase in the number of reports of tumor-on-chip devices, an extensive overview of which is available by Li et al. (2018). Microfluidic chips have been applied in BC diagnostics for quick and standardized detection of specific biomarkers like: ER, PR, HER2, and Ki67 (proliferation marker) on fixed tissue samples (Cho et al., 2018; Aimi et al., 2019), and for early detection of circulating cancer cells (Fang et al., 2017). Microfluidic diagnostics and novel 3D models provide insight into the physiology of patient-specific BCs, open the door to precision medicine, and have already been used to test new and alternative treatments for BCs (Cho et al., 2018).

Microfluidic chips are fabricated using a variety of techniques (soft lithography, embossing, and other relief methods for generating negative molds) and with different materials (primarily polymeric). Applications in cell biology often employ polydimethylsiloxane (PDMS), due to its favorable properties including: optical transparency, tunable range of stiffness, low toxicity, chemical inertness, gaseous permeability, and its relatively low cost (Fiorini and Chiu, 2005). One major limitation of PDMS is that it absorbs small molecules, and so it is not appropriate for long-term drug studies (Fiorini and Chiu, 2005). Aa scaffold-based 3D models, microfluidic chips are typically used to structurally confine cells in either natural or synthetic hydrogels, which are useful for recreating a natural-like tissue environment due to their amenable and tunable properties (Koo and Velev, 2017). These technologies are apt for reproducing the complex tissue environment of breast cancers, a number of which are outlined in Table 1 and will be explored in the next subsections.

**TABLE 1:** Classification of the different publications on breast tumor-on-chip devices.

BC subtype	BC cell line	Stromal cells	Perfusable vessels	Immune cells	Native matrix	Synthetic matrix	Treatment	References
DCIS	MCF10-DCIS	HMF	X	X	Mat, Col I	X	X	Sung et al., 2011
DCIS	MCF10-DCIS	MCF10a HMF	X	X	Mat, Col I	X	X	Bischel et al., 2015
DCIS	MCF10-DCIS	HMT3522-S1; HMF	X	X	Col I	X	Paclitaxel	Choi et al., 2015
DCIS	MCF10-DCIS	MCF10a HMF	X	X	Col I	X	Tirapazamine Doxorubicin	Ayuso et al., 2018
Luminal-A	T47D	HMF	X	X	ECM gels	X	X	Montanez-Sauri et al., 2013
Luminal-A	T47D	HMVEC	✓	X	X	PuraMatrix hygrogel	Tarceva, staurosporine,	Dereli-Korkut et al., 2014
Luminal-A	MCF7	NMF; CAF	X	X	X	PCL	X	Gioiella et al., 2016
Luminal-A	MCF7	HUVEC, hLF	✓	X	FN	X	X	Nashimoto et al., 2017
Luminal-A,TNBC	MCF7,MBA-MD-231	HUVEC	X	X	X	GelMA	Doxorubicin	Aung et al., 2016
Luminal-A,TNBC	MCF7, MBA-MD-231	HUVEC	X	THP-1 TALL-104	X	GelMA	X	Aung et al., 2020
Luminal-A,TNBC	MCF7, MBA-MD-231	HBTAEC	✓	X	Mat	X	Liposomal drug carriers	Tang et al., 2017
Luminal-A,TNBC	MCF7, MBA-MD-231	HUVEC	✓	X	FN	X	X	Nagaraju et al., 2018
Luminal-A,TNBC	MCF7, MBA-MD-231	ECFC-ECs, NHLF	✓	X	FN	X	5-Fluoruracil, vincristine, Sorafenib taxol	Sobrino et al., 2016
Luminal-A,TNBC	MCF7, MBA-MD-231	hBTEC,BJ-5ta	✓	X	X	PF-Hydrogel	Doxorubicin paclitaxel	Pradhan et al., 2018
Luminal-A,TNBC	MCF7, MBA-MD-231, PDTO	ECFC-ECs, NHLF, NBF, CAF	✓	X	FN	X	Paclitaxel	Shirure et al., 2018
Luminal-A,TNBC	MCF7, MBA-MD-231	HUVEC, NHFL, MCF10a	✓	X	FN	X	X	Song et al., 2018
Luminal-A,TNBC	MCF7, MBA-MD-231	HUVEC, NHFL	✓	X	FN	X	Paclitaxel	Nashimoto et al., 2020
Luminal-A,TNBC	T47D,BT549	HUVEC	✓	X	BME Hydrogel	X	Doxorubicin	Chen et al., 2018
Luminal-A,TNBC	MCF7, MBA-MD-231	HUVEC	✓	X	Col I	X	AMD3100	Kong et al., 2016
TNBC	MBA-MD-231	HUVEC, hBM-MSCs,OD- hBM-MSCs	✓	X	FN	X	X	Jeon et al., 2015
TNBC	MBA-MD-231	HUVEC, hBM-MSCs,OD- hBM-MSCs	✓	X	Col I	X	X	Bersini et al., 2018
TNBC	MBA-MD-231	TIME	✓	X	Col I	X	X	Michna et al., 2018
TNBC	MBA-MD-231	HUVEC,NHFL	✓	Patient-derived monocytes	FN	X	IIA-inhibitor blebbistatin	Boussommier-Calleja et al., 2019
TNBC	MBA-MD-231	HUVEC,MCF10a	X	TAM, U937	Col I	X	Paclitaxel	Mi et al., 2019

*Groupings are based on the different breast cancer subtypes and the specific cell lines used the stromal cells and the immune cells employed, the presence of a perfusable vasculature in the system, the use of synthetic or native matrix and if specific BC treatment are used in the system. The following abbreviations are reported as: Mat, Matrigel; Col I, Collagen I; FN, Fibrinogen hydrogel; PCL, polycaprolactone; GelMa, gelatin methacryloyl.*

#### Tumor-on-Chip Devices of Ductal Carcinoma *in situ*

**FIGURE 2:**
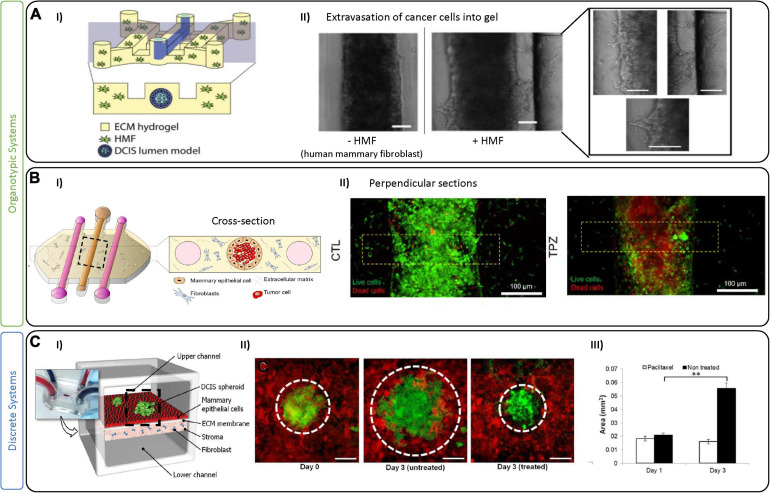
Tumor-on-chip devices for ductal *in situ* carcinoma. **(A)** Example of fibroblast-associated DCIS migration. **(I)** Schematic representation of the tumor-on-chip device. The central lumen is lined with MCF-10 (normal epithelial cells) to mimic a mammary duct and then seeded with cancer cells (MCF10aDCIS). Fibroblasts are added in a collagen I hydrogel to adjacent chambers. **(II)** Bright-field images show the mammary duct filled with DCIS cells either alone or in co-culture with fibroblasts (HMF), which promote an invasion (as seen in right most images). Scale bars are 100 μm. Figures are adapted from Bischel et al. (2015). **(B/C)** Example of a 3D DCIS lumen model with additional perfusion channels. (I) Schematic of the device with three channels generated by removable PDMS rods that create a centrally lined lumen (MCF10 cells) and 2 flanking lumens to perfuse media, metabolites or drugs. Similar to the model in **(A)**, the central lumen is filled with DCIS cancer cells and fibroblasts in the surrounding hydrogel. (II) Live/dead images showing control and Tirapazamine (TPZ) treated DCIS in the lumen. After 3 days in culture, cell viability was evaluated demonstrating TPZ-associated toxicity in the center (hypoxic region) of the lumen. Figures adapted from Ayuso et al. (2018). **(C)** An example of a multi-layer perfusable DCIS model. **(I)** Schematic figure showing compartmentalized DCIS spheroids on top of a mammary epithelial layer with fibroblasts in a collagen gel in a bottom layer. **(II)** DCIS spheroids at day 0, and day 3 with and without paclitaxel. **(III)** Paclitaxel treatment was shown to prevent further growth of the DCIS spheroids in this model. Scale bars are100 μm. ^∗∗^*p* < 0.05. Figures are adapted from Choi et al. (2015).

Abundant evidence suggests that the TME surrounding the DCIS regulates and promotes BC conversion to an invasive phenotype (Nelson et al., 2018). Of the most frequently studied cell lines, MCF-10 (a non-cancerous breast epithelial cell line) and MCF10-DCIS (for DCIS) were developed at the Michigan Cancer Foundation and have allowed extensive study into this BC subtype (Soule et al., 1973, 1990). In DCIS, cancer cells are separated by the physical barrier of the mammary duct, and microfluidic devices have been designed to represent this separation from the stroma. For example, Bischel et al. recreated a mammary duct by lining MCF-10 (non-tumoral) cells in a lumen formed by a hydrogel containing fibroblasts (Figure 2A). Epithelial mammary cells formed a monolayer similar to an *in vivo* duct, following which, DCIS cells were seeded inside the lumen. This model demonstrated that paracrine signaling alone from fibroblasts can cause DCIS cell invasion into the surrounding ECM (Bischel et al., 2015). Similarly, Sung and colleagues used a two-channel microfluidic device to capture paracrine and juxtracrine signaling events between fibroblasts and DCIS cells. While fibroblasts were seen to promote morphological changes in the cancer cells through paracrine signaling, a complete transition to an invasive phenotype only occurred when in direct contact (Sung et al., 2011). Given the conflicting findings of these studies and the indication of the role of the TME in tumor progression (Nelson et al., 2018), it will be crucial to investigate these complex interactions in the DCIS further using similar models. Models of DCIS have also been proven useful to test drugs targeting components of the TME. As an example, Ayuso et al. (2018) generated multiple lumen structures using removable PDMS rods (Figure 2B) to generate a mammary duct cultured with MCF-10 cells surrounded by ECM and fibroblasts. The advantage of their system is the inclusion of adjacent lumen to perfuse media and drugs, and to collect supernatant for downstream analysis. Their system was used to investigate hypoxia, nutrient depletion and oxidative stress, which promoted an invasive tumoral phenotype, confirmed by evaluating metabolic and gene expression changes (Jimenez-Torres et al., 2016; Ayuso et al., 2018). They also employed Tirapazamine (TPZ), an anticancer drug activated by low levels of oxygen, to specifically attack hypoxic tumor cells in their model. TPZ had no effect at the periphery of the ductal lumen, or on the surrounding fibroblasts, but it did reach the core of the breast lumen and was effective at killing the resident hypoxic cells. In another example, Choi et al. developed a compartmentalized 3D platform allowing for culture of human primary fibroblasts in a lower chamber with epithelial cells and MCF10-DCIS spheroids in an upper chamber (Figure 2C; Choi et al., 2015). The effect of a constant flux of paclitaxel (a tubulin-targeting drug) was shown to slightly decrease the growth of the DCIS tumor spheroids. Although a step in the right direction, their system relies on a homogenous distribution of drugs supplied to the epithelial layer. Pharmacokinetics of drug transport will rely on complex interactions with the circulatory system, as well as other stromal and TME components, and could be implemented in the future. The results of these studies underline the importance of considering the TME in model development; however, many players are yet to be included (myoepithelial cells, adipose cells, myeloid and immune cells), as well as the intra-tumoral vessels that are not considered in any of the current models of DCIS. On one hand, adding complexities (cell types, relevant geometries, etc.) to models can enhance their physiologic relevance; however, they will also complicate individual effects on tumor growth and suppression.

#### Tumor-on-Chip Devices of Luminal-Like BC

**FIGURE 3:**
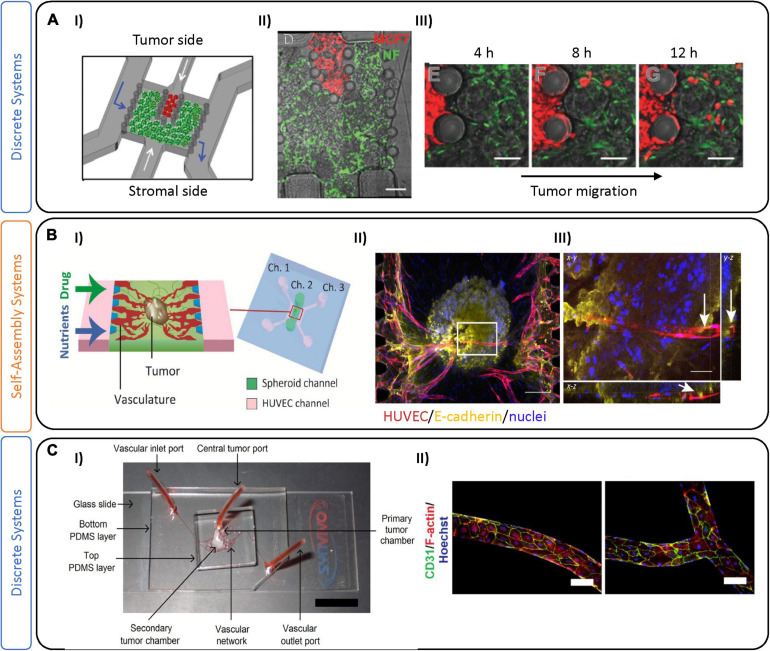
Tumor-on-chip devices for luminal-like breast cancer. **(A)** Example of a luminal-like model using physical separation on-chip to analyze tumor migration. **(I)** Schematic representation of the tumor-on-chip device, composed of two compartments for tumor cells (red) and stromal cells. The chambers are separated by interspaced pillars that allow physical contact between tumor and stromal compartments. **(II)** Fluorescent image of the device with normal fibroblasts expressing GFP and MCF7 cells expressing RFP. **(III)** Time sequence showing cancer cell migration and invasion. Scale bars are 100 μm. Adapted from Gioiella et al. (2016). **(B)** Microfluidic device leveraging self-assembly on-chip to investigate sprouting angiogenesis toward a solid breast tumor. **(I)** This chip is characterized by a central channel encompassing an MCF-7 and fibroblast spheroid, with two lateral channels (Ch.1 and Ch.3) containing endothelial cells (HUVEC) and media. **(II)** Immunofluorescence images of the tumor spheroid with innervating vasculature. Scale bar is 200 μm. **(III)** Projection image of the tumor spheroid demonstrating its vascularization (indicated by white arrows). Scale bar is 50 μm. Figures adapted from Nashimoto et al. (2020). **(C)** Microfluidic device incorporating pre-defined vascular geometry to generate regions of high and low shear flow. **(I)** Three-layer PDMS microfluidic device where MCF-7 and MBA-MD-231 BC cells were maintained in long-term 3D co-culture with stromal fibroblasts in a poly(ethylene glycol)-fibrinogen hydrogel matrix within adjoining tissue chambers. The central tumor port is located on the top PDMS layer and is directly connected to the primary tumor chamber via a vertical channel of diameter 0.75 mm. Vascular inlet and outlet ports on the bottom PDMS layer facilitate the flow of media, reagents, and seeding of endothelial cells. **(II)** Fluorescence images of the vasculature in the pre-defined geometries which were seeded with human breast tumor-associated endothelial cells (hBTECs) and maintained under flow in various sections of the microfluidic channels. Figures adapted from Pradhan et al. (2018).

The most frequent subtype of BC, luminal-like, is specifically identified by high expression levels of ER, PR, and HER2 receptors (Eliyatkin et al., 2015). Patients with this tumor type undergo surgery and chemotherapy and/or radiotherapy (depending on the tumor features) and can also benefit from long-term endocrine or targeted therapies to prevent tumor relapse. Due to its treatability, personalized treatments could be considered to omit chemotherapy (often an adjuvant therapy) (Fragomeni et al., 2018). Other targets, including those of the TME, could be used as alternative therapies, but a reliable preclinical model is still required to test them (Montanez-Sauri et al., 2013; Aung et al., 2016). As an example, the microfluidic device developed by Gioiella et al. (2016) used MCF-7 luminal-like cancer cells, fibroblasts, and endothelial cells, to characterize stromal cell activation (Figure 3A). Their device contains a central tumor cell compartment surrounded by a stromal compartment, which is separated by an interface that allows for physical contact. The authors demonstrated the activation of fibroblasts into myofibroblasts, induced by the co-culture via immunofluorescent characterization of αSMA and platelet derived growth factor (Gioiella et al., 2016).

Microfluidic devices represent a powerful tool to investigate the role of the vasculature (Haase and Kamm, 2017), which can be implemented in models of tumorigenesis. Several groups have recently formed perfusable microvessels by seeding combinations of endothelial cells and fibroblasts embedded in hydrogels together with tumoral cells (Sobrino et al., 2016; Agarwal et al., 2017; Chung et al., 2017; Mannino et al., 2017; Nashimoto et al., 2017; Tang et al., 2017; Song et al., 2018; Xiao et al., 2019; Haase et al., 2020). For instance, Nashimoto et al. demonstrated vascularization of MCF-7 and lung fibroblast spheroids by co-culture with human umbilical vein endothelial cells (HUVEC), showing angiogenic sprouting toward the tumor (Figure 3B; Nashimoto et al., 2020). Drug delivery via their perfusable vasculature demonstrated that, under perfusion, dose-dependent effects of chemotherapeutics were abrogated, in contrast to static conditions. Pradhan and colleagues also demonstrated reduced cytotoxic effects under vascular luminal flow, compared with static culture (Figure 3C; Pradhan et al., 2018). Instead of relying on endothelial cell self-assembly, their model geometrically defined vasculature using monolayers of tumor-associated breast endothelial cells (vessels) to recreate areas with high and low perfusion, to mimic heterogeneous tumor perfusion in a controlled manner. Co-cultures of MCF-7 and fibroblasts in the tumor region allowed for long-term culture and monitoring for up to 28 days, that showed the development of a central necrotic core. We note in this example, and others (Pradhan et al., 2018; Nashimoto et al., 2020), that the presence of a central necrotic core is often used to suggest similarity to tumors *in vivo*. The presence of a necrotic core is not typically characteristic of early stage breast tumors; however, necrotic foci are often present in late-stage solid tumors with a highly metastatic potential (Jiao et al., 2018). The molecular characteristics *in vivo* involve complex pathways (including necroptosis) that are not yet fully understood, and are likely quite disparate from the necrotic core that develops *in vitro*. The real strength of this study was to examine proliferation of tumor cells in close contact with vasculature, thus recapitulating some aspects of *in vivo* drug delivery. Whether through self-assembly, or through pre-defined geometry, these studies underline the importance of incorporating tumor-associated vasculature in BC models, which allows for more accurate drug delivery and altered drug response, and likely will increase the predictive capacity of these models. Recently, we have also generated vessels using a vasculogenesis-like process that relies on self-assembly of endothelial cells to form vessels. We have used this technique to examine drug interactions on non-BC tumor spheroids and show that they are largely different from drug interactions in tumor spheroids (without a TME) (Haase et al., 2019). Work in our laboratory is now focused on using these techniques applied specifically to BCs.

#### Tumor-on-Chip Devices for Triple Negative Breast Cancer

The TME of TNBCs has been proposed to contribute to its high propensity for metastasis. For instance, TNBCs are more likely to demonstrate lymphocyte infiltration (up to three times higher) compared to other BC subtypes, and they are associated with a higher number of infiltrated TAMs, in particular the M2 subtype (Disis and Stanton, 2015; Stanton and Disis, 2016). TNBC patient biopsies demonstrated that stroma-low tumors have better outcomes in the 5-year relapse-free period and overall better survival. Interestingly, patients with TNBC have significantly higher intra-tumoral VEGF levels, which enhances the ability of endothelial cells to form vascular-like channels (Ribatti et al., 2016). A pro-angiogenic TME could indeed lead to increased tumor vessel co-option and metastases. The unique TNBC microenvironment highlights the importance of generating appropriate models for its study, and could imply TME components as potential targets for future treatment. Given the severity of TNBC, studies have focused on specific aspects of the disease, including tumor-immune interactions and metastasis, several examples of which will be further discussed below.

In TNBCs, the immune system is highly attracted to the tumor site due to genome instability of the cancer cells and the expression of pro-tumorigenic and chemoattractant factors (Binnewies et al., 2018). While the role of lymphocytes in TNBC has been largely studied, other immune cell interactions are less well-known, and have been reported as both pro- and anti-tumorigenic. For example, monocytes contribute to both pro- and anti-tumoral immunity, recruitment of lymphocytes, and differentiation into tumor-associated macrophages and dendritic cells (Allaoui et al., 2016). Controlling monocyte plasticity and heterogeneity could potentially serve as a new therapy against TNBC.

Several recently published models outline various strategies for investigating BC tumor-immune cell interactions. In a recent example, Aung et al. (2020) demonstrated a hydrogel encapsulation approach to integrate monocytes, MDA-MB-231, and HUVEC. Photopatterning was used to encapsulate monocytes and a TNBC spheroid in a central hydrogel core, which is surrounded by a second hydrogel containing a continuous layer of endothelial cells to mimic the blood vessel wall. The presence of monocytes in this system demonstrated enhanced intravasation and migration of T-cells toward the TNBC spheroid via the endothelial layer. Chemokines released by monocytes effectively decreased the barrier function of the endothelial cells, enabling intravasation of the T-cells (Aung et al., 2020). This work clearly demonstrates monocyte-mediated tumoral T-cell attraction; however, some aspects of the TME—such as the monolayer to represent vasculature, are simplified and could affect the results, particularly given that significant differences in barrier function have been shown between 2D and 3D *in vitro* vessels (Offeddu et al., 2019).

**FIGURE 4:**
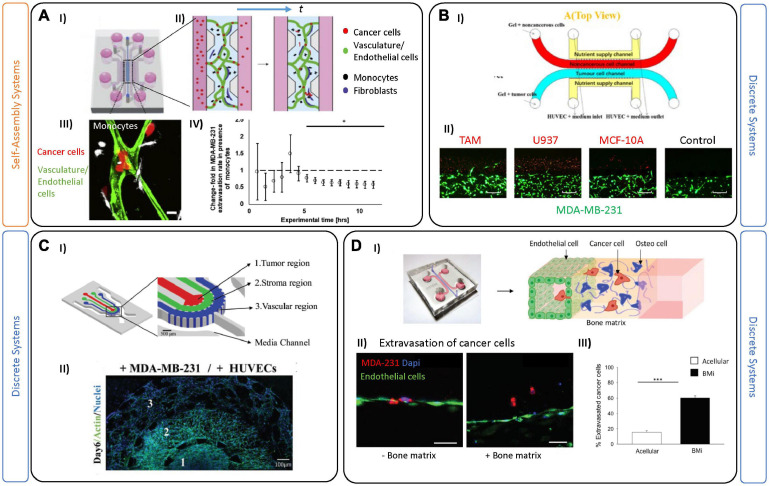
Tumor-on-chip devices for triple negative breast cancer. **(A)** Example of self-assembled vasculature on-chip to investigate complex tumor-immune interactions. **(I)** PDMS platform encompasses a central chamber filled with fibrin hydrogel and a mix of endothelial cells and fibroblasts. **(II)** Experiments were performed by perfusing vessels on-chip with monocytes (black) and tumor cells (red). **(III)** A corresponding confocal image demonstrates tumor-immune cell interactions in both intra- and extra-vascular regions. In these experiments, monocytes were first perfused and extravasated across the endothelial barrier followed by tumor cell perfusion 2 days later. Scale bar is 10 mm. **(IV)** Quantification of the extravasation rate of MDA-MB-231 tumor cells decreases after 5 h in the presence of monocytes in the system. Figures adapted from Boussommier-Calleja et al. (2019). *P < 0.5. **(B)** Two-gel system for investigating tumor-TME associated migration patterns. **(I)** Schematic representation showing a top (red) channel filled with macrophages and a bottom (blue) channel filled with tumor cells. **(II)** Images demonstrate invasion of breast cancer cells MDA-MB-231 cocultured with different non-cancerous cells (in red) like tumor associated macrophages (TAM); U937 normal macrophages, MCF-10a and a blank control group (no migration of tumor cells). Scale bars are 500 mm. Figures are adapted from Mi et al. (2019). **(C)** Example of on-chip TME partial-confinement for examining TNBCs. **(I)** Schematic representation showing three regions partially connected: tumor, stroma, and the vascular regions. The regions are distinct but allow for diffusion of biomolecules and dynamic heterotypic interactions at the interfaces (including migration of tumor cells). **(II)** Representative fluorescence image of the entire culture region on day 6. Figures adapted from Nagaraju et al. (2018). **(D)** Microfluidic single-gel device used to mimic extravasation of TNBC. **(I)** Image and schematic showing the extravasation process of cancer cells from the endothelial barrier (HUVEC) toward a bone tissue microenvironment. **(II)** Representative images of the MDA-MB-231 extravasated into the extracellular matrix in acellular condition or bone microenvironment condition. Endothelial layer cancer cells (red), cell nuclei (blue). Scale bars are 50 mm. **(III)** Bar plots demonstrate extravasated cancer cells in either acellular or bone microenvironments (mean SEM). Figures adapted from Bersini et al. (2018). ***P ≤ 0.001.

As mentioned earlier, several groups have focused on generating complex *in vitro* vascular systems, comprised of perfusable interconnected vessels which maintain functional barrier properties similar to those seen *in vivo* (Haase and Kamm, 2017). By taking advantage of the natural ability of endothelial cells to self-assemble, Boussommier-Calleja et al. (2019) perfused monocytes through an on-chip luminal vascular network. After several hours post-perfusion, most of the monocytes were trapped inside the luminal vessels. A decrease in the extravasation efficiency of TNBCs was shown due to the presence of paracrine signals, corresponding with an anti-metastatic role of monocytes (Figure 4A; Boussommier-Calleja et al., 2019). In contrast, Mi et al. (2019) created an invasion system with two compartmentalized gel channels: one seeded with MBA-MD-231 cells, and the other with macrophages and an endothelial barrier (Figure 4B). In their model, TNBC cells in contact with TAMs led to an invasive phenotype, as demonstrated by increased survival of tumor cells following paclitaxel treatment. As they demonstrated, TAMs can potentially aid in drug resistance, thus targeting the immune system may be useful as a BC therapy—one that can be examined in systems such as those outlined.

Besides a strong immune-response, TNBC is the most prone BC subtype to metastasize. Phases of the metastatic process have been extensively studied and described (Tarin, 2011), and briefly include: detachment of cancer cells from the primary tumor, subsequent intravasation into the vasculature, circulation in the blood stream, and finally extravasation and colonization in a distant organ (Seyfried and Huysentruyt, 2013). So far, different aspects of BC metastasis have been investigated using different *in vitro* tools. For instance, 2D assays using transwell chambers have been employed to examine migration of cancer cells in the presence of chemotactic gradients, mimicking the microenvironment of the guest organ (Katt et al., 2016). Microfluidic devices are also primed for investigating extravasation across the endothelial barrier and to distant regions (Crippa et al., 2021). For example, Nagaraju et al. (2018), employed a microfluidic device with three distinct compartments to track migration of circulating tumor cells (CTCs) into a stromal compartment, and subsequently into a vascular one (Figure 4C). The cancer cells secreted several biochemical factors (including VEGF) which modified the vascular networks, as seen by decreased diameter and increased vessel leakiness (Nagaraju et al., 2018). By weakening the endothelial barrier, the tumor cells can enter the circulatory system (extravasation). Most circulating tumor cells die, likely due to a combination of physical and oxidative stress; however, CTCs that survive exit the bloodstream and begin to divide and colonize a new organ (Micalizzi et al., 2017). Several studies have used microfluidic chips to recreate this final phase of metastasis—by tracking CTCs crossing the endothelial cell layer under luminal flow. Importantly, these studies demonstrated that inflammatory molecules, including CXCL12, attract and promote the extravasation of cancer cells (Song et al., 2009; Chen et al., 2013, 2018; Michna et al., 2018; Sleeboom et al., 2018; Toh et al., 2018).

For BC, metastasis usually occurs in bone, liver, lung, and brain, and is hypothesized, in-part, to be influenced by organ-specific microenvironmental cues. Interestingly, BC subtypes present unique tissue preferences, as shown by luminal-like BCs which are more prone to metastasize in bone, while basal-like BCs tend to prefer visceral organs and soft tissue, including lung and brain (Dent et al., 2009). Metastasis to bone makes up 70% of these cases, and it is still unclear which aspect of this tissue may contribute to it being a preferred target (Pulido et al., 2017). Many transwell-based assays have been used to investigate chemotaxis of BCs and potential intravasation of CTCs toward a potential guest organ (Liu et al., 2019). While useful, monolayer-based approaches cannot achieve the complexity (dynamic flow-mediated vessel adhesion) of multi-organs-on-chip, which have recently been employed to examine simultaneous potential for metastases to various organs (Xu et al., 2016; Liu et al., 2019). For example, a recent multi-layer device was designed to model CTC circulation and intravasation from HUVEC-lined channels toward compartments cultured with primary cells from muscle, lung, liver and bone (Kong et al., 2016). The study demonstrated the preferential metastasis of MCF7 and MDA-MB-231 to lung, which was confirmed in a mouse study. Other multi-organ metastases models have also been employed for other cancers, including lung (Ozkan et al., 2019; Tian et al., 2020b). Several bone-on-chip devices have been developed to understand the bone-ECM and tumor cell interactions (Bersini et al., 2014; Jeon et al., 2015; Hao et al., 2018; Marturano-Kruik et al., 2018; Mei et al., 2019). As an example, Bersini et al. (2018) injected TNBC cells into an endothelial-lined channel, and observed that the presence of an adjacent bone-mimicking microenvironment (composed of osteo-derived cells and bone-matrix component) an increased extravasation of the CTCs, compared to an acellular system (Figure 4D). Transcriptomic analysis of extravasated cancer cells was performed and suggested an upregulation of proteases (MMP, a-disintegrin) involved in endothelial glycocalyx shedding. Coupling of transcriptomics analysis to extravasated cells would be impossible to perform in animal models, again highlighting a major advantage of using 3D culture systems.

## Challenges and Future Perspective of Breast Tumor-On-Chip Models

Tumor-on-chip systems are increasingly employed to model breast cancer; however, we are still at the beginning stage of developing subtype-specific varieties of this widespread disease. Tools including microfluidics-based models, as shown, can provide control over the TME to generate relevant 3D culture platforms for BCs. The TME, as discussed, plays a significant role in breast tumor development and progression and these devices can be designed to model particular aspects of the disease. For instance, for DCIS several models present organotypic systems—mimicking the physical breast lumen structure where the tumor develops. These models are suitable for mimicking tumor growth and cell migration from the duct. However, other models employ physical separation of TME components on-chip (discrete systems) in order to investigate tumor-TME interactions in a more defined manner. For instance, luminal and TNBC models have used this approach (physical separation) to interrogate how the different TME components (stromal cells, vasculature, immune system, circulating cancer cells, etc.) interact and influence tumor development. Recently, several groups have also used self-assembly approaches to model different components of the TME (vasculature and stroma) together with cancer cells. These self-assembly systems are arguably more realistic for modeling particular aspects (extravasation, tumor cell infiltration, etc.) of tumor development and recapitulate the process in a more physiologic way, but are more difficult to assess and quantify.

Strategies to generate organ-like features, or to integrate TME components in physically constrained or heterogenous manners, are useful for understanding particular aspects of BC development. However, these artificial *in vitro* microenvironments are still far from the complexity of the *in vivo* situation. The minimum number of essential elements required to generate an ideal representative BC model remains an open question. Multi-cell cultures on-chip, to the best of our knowledge, have so-far been limited to combinations of endothelial cells, fibroblasts, cancer cells and immune cells. Since each breast-specific cell type requires specific culture conditions, it is difficult to maintain (via compromises in media selection) viable complex cultures *in vitro*. Another complication is the use of serum in most culture systems, which leads to a lack of reproducibility and standardization, which is essential for drug screens. Thus far, most models lack interactions with adipocytes and myoepithelial cells—potentially due to their complex culture conditions (Artacho-Cordon et al., 2012); however, inclusion of these cell types would significantly enhance the physiologic likeness of the BC microenvironment. From a reductionist point of view, additional elements may increase the physiologic relevance, but will add complexity in maintenance and characterization of these models. Nevertheless, these models will require essential features to accurately demonstrate human drug interactions.

Most breast cancer on-chip models discussed employ established and well-characterized immortalized cell lines, which, due to their ease of culture, are extremely useful and reproducible across research groups. TNBC is the most commonly studied BC on-chip because of the variety of available cell lines. One particular cell line, MBA-MD-231, is the most common, and while it is ER, PR, and HER2 negative, it also expresses mutated p53 (which is common in 80% of TNBCs) and a gene expression profile of a basal-like TNBC (Cailleau et al., 1974). With this in mind, it is important that cell lines and the questions they aim to address are chosen carefully. The study of Luminal-A subtype is, in large part, due to widespread use of MCF-7; a cell line that is easy to culture and forms tumor spheroids (Froehlich et al., 2016); in contrast, the lack of suitable Luminal-B cell line has limited its study, despite its poorer prognosis (Haque et al., 2012). Lately, a large number of breast cancer cell lines have been made available via biobanks and there has been a huge effort to sequence and properly classify these subtypes (Bruna et al., 2016). Despite the variety available, two-thirds of BC research are based on the use of 3 common cell lines (MBA-MD-231,MCF-7 and T47D) making it clear for the need to encourage the use of lesser studied and variable subtypes (Dai et al., 2017).

**FIGURE 5:**
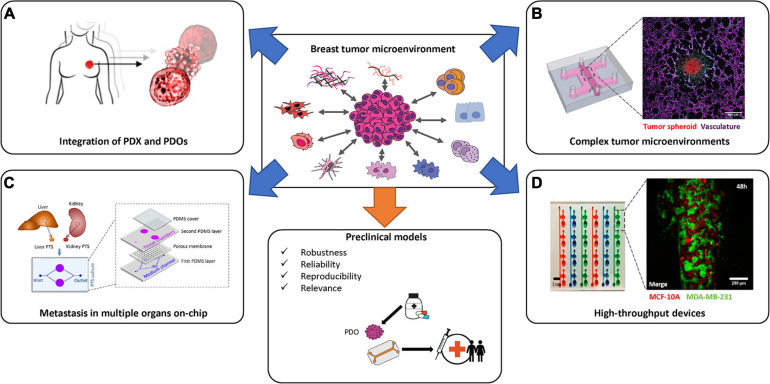
State-of-the-art of breast cancer *in vitro* models and reaching preclinical use. Several strategies have been employed in order to generate novel *in vitro* breast cancer specific models. Complex systems that combine appropriate TMEs with patient derived organoids (PDOs), in a high-throughput manner, will be necessary to recapitulate the complexities of breast cancer subtypes prior to the generation of robust preclinical tools. Images in **(A)** are adapted from Sachs et al. (2018). Images in **(B)** (left-most image) is adapted from Haase et al. (2020) and the (right-most image is by the author K. Haase). Image in **(C)** is from Tian et al. (2020b). Image in **(D)** is adapted from Virumbrales-Munoz et al. (2019).

On the other hand, advantages of incorporating patient-derived samples on-chip includes preservation of native architecture and cross-talk between different tumoral cell types. Moreover, coupling patient-samples in breast cancer on-chip models could open new doors for personalized treatments (Sachs et al., 2018). Still, obtaining fresh tissue samples from a BC surgery or biopsy is challenging and often results in small numbers of cells which cannot be maintained easily, or lose their phenotype in *ex vivo* culture conditions. Limited growth of primary cells, and variability in establishment and differentiation of iPSCs, respectively, have limited the use of patient samples in breast cancer on-chip devices (Shirure et al., 2018; Truong et al., 2019; Figure 5A).

Models to-date still fail to represent the complex, dynamic, conditions *in vivo*, nor the true physical likeness of mammary tissue or its components. Future studies should incorporate the use of pumps, for example, to control nutrients and/or chemokine gradients, as well as mechanical cues associated with fluid flow. Other mechanical cues (i.e., pressure gradients or stretch) can also be mimicked on-chip, and have thus far been lacking in breast tumor-on-chip models. For example, ovarian cancer cells cultured in a microfluidic device under fluid shear stress resulted in enrichment of the cancer stem cell population and enhanced tumor chemoresistance to anti-cancer drugs (Ip et al., 2016). Biochemical and mechanical cues can alter the behavior, patterning, genetic and epigenetic landscapes, and thus should provide insight into BC behavior when controlled on-chip (Wang et al., 2018).

Another important factor to consider is the different physical design of these systems. For DCIS studies, most publications report a duct-like physiology in their platform. For other BC subtypes, the microfluidic devices presented herein, demonstrate highly varied and sometimes non-physiologic geometries. Each microfluidic device is typically designed to address a specific biological question and may not provide a complete picture of tumor behavior as it would be in the breast tissue. For example, in Figure 3B, the device is optimized to show angiogenic sprouting toward the tumor spheroids (Nashimoto et al., 2020). Some groups, including our own, are focusing on developing vascularized tumor models to study drug delivery by perfusion through a vascular network to solid tumors (Haase et al., 2020; Figure 5B). These models still do not incorporate all aspects of breast tissue, which would entail ducts and/or lobule likeness and fatty tissue. Reproducing the breast gland in its entirety on-chip will pose a challenge, given the many cell types, tissue and stimuli that are physiologically present in the breast. Despite not yet reaching this goal, current models have been quite effective at dissecting specific BC events, and have provided increased understanding of complex tumor growth and suppression.

Incorporating tumor cell dynamics on-chip is important, particularly given the role the vascular system plays in metastasis. For complete metastasis to occur, tumor cells must first intravasate through the basement membrane and squeeze through the endothelial barrier, then they must survive the blood circulation, before finally extravasating through attachment and migration into distal tissues (Deepak et al., 2020). A significant number of BC patients (33.07%) develop multiple-site metastases, which, depending on site, can be associated with a poorer prognosis (Wang et al., 2019). Research groups have developed and used multi-organ-on-chip devices to recreate multi-site metastasis. For instance, breast and liver tissues have been coupled (Ozkan et al., 2019), as well as bone and liver on-chip (Kong et al., 2016). In one example, Tian and colleagues employ primary mouse tissue biopsies to generate a liver—kidney connection on-chip (Figure 5C; Tian et al., 2020b). Extracellular vesicles produced by BC cells were introduced into the system and demonstrated that CXCL12/CXCR4 participated in liver tropism (increased uptake occurred in liver, in contrast to kidney) (Tian et al., 2020b). Extracellular vesicles have been implicated in the development of a pre-metastatic niche, which may facilitate cancer cell colonization and are more easily assessed on-chip than *in vivo*. Multi-organ-on-chip systems can reveal important information about secondary metastasis and if connected via vascular networks could help to recapitulate characterization of CTCs *en route*, or simply could help to define toxicity of the treatment in secondary organs like in the case of the study of Lee and colleagues (Lee et al., 2021).

For examining existing or new drug panels, 3D *in vitro* models need to be high-throughput, robust, and generate reproducible results. Several companies like Mimetas, Synvivo, 4Design Biosciences and AIM Biotech have each commercialized 3D culture plates, largely based on microfluidic technologies, and use alternative materials to PDMS that support several organ-on-chip devices in a single platform. Most companies offer custom designs of their platform for specific applications, and several have been implemented for BC studies (Lanz et al., 2017; Virumbrales-Munoz et al., 2019). These multi-chip platforms need further development for use with BC specific subtypes and patient samples, in order to be used as promising tools for BC research and diagnoses (Figure 5D). Integration of the aforementioned systems with iPSCs derived from PDOs, could lead to completely integrated complex tumors-microenvironments on-chip, useful for precision medicine. Due to the limited sample size required, tunable microenvironment and low overall costs, tumor-on-chip models have the benefit of being able to model rare disease, examine off-target effects of treatment (to vessels, for example) and have the potential to be adopted for preclinical breast cancer research. Breast-specific factors, including sex hormones, should be considered during model development, as they strongly influence the tumor microenvironment and the response to treatment. It is imperative to collect quantitative data that relates the tumor on-chip to clinical data, and thus *in vivo* translation and computational models are still valuable intermediary steps in developing preclinical tools. Test sites have been established in the US to provide analytical validation of these tissue-on-chip systems to assess robustness, reliability, reproducibility and relevance to standardized compounds, assays and biomarkers, as advised by the FDA (Tagle, 2019). These rigorous tests and public-private partnerships are needed worldwide to establish useful and standardized preclinical *in vitro* tools, and will benefit greatly the translation of BC microfluidic models to the clinic.

## Conclusion

Breast cancer research is an active field of study, complicated by its various subtypes and classifications. Although simpler 2D assays and complex animal models have provided significant insight into cancer development and progression to date, there remains a lack of well-established *in vitro* 3D assays specific to breast cancer subtypes. Novel microfluidic technologies now importantly incorporate key features of the breast tumor microenvironment, allowing for novel targets to be examined. For instance, tumor-on-chip models integrating functional vasculature can be employed to study complex immune interactions and metastasis—both of which are key to understanding triple negative breast cancers. Integration of patient cells, along with the development of adaptive high-throughput models, will improve breast cancer treatment specificity and allow for *in vitro* tool adoption for personalized medicine. The models reported to-date have revealed critical insight into breast cancer biology, yet they are still in their infancy and require further development prior to implementation as preclinical tools. The main challenge remains to demonstrate the accuracy of these models in predicting the human response to existing and novel drugs. To do so, bioengineers and research scientists need to collaborate with clinicians and biobanks to test large numbers of patient samples using well-defined robust subtype-specific models, which will promote their utility as preclinical tools.

## Author Contributions

CM reviewed the literature, wrote the manuscript, and designed the figures. KH supervised the work and contributed to writing and editing the manuscript. Both authors contributed to the article and approved the submitted version.

## Conflict of Interest

The authors declare that the research was conducted in the absence of any commercial or financial relationships that could be construed as a potential conflict of interest.
